# Utilisation of intramuscular and intermuscular fat to develop a new skeletal muscle grading score which can predict treatment outcomes for locally advanced rectal cancer

**DOI:** 10.1007/s00384-026-05106-w

**Published:** 2026-02-16

**Authors:** Alex Besson, Ke Cao, Michael Rouse, Josephine Yeung, Fiona Reid, Peter Gibbs, Justin M. Yeung

**Affiliations:** 1 Department of Surgery, Western Precinct, The University of Melbourne, Melbourne, Victoria, Australia; 2 Melbourne Academic Centre for Health, North Melbourne, Victoria, Australia; 3 Department of Colorectal Surgery, Western Health, Footscray Hospital, Melbourne, Victoria, Australia; 4 Walter and Eliza Hall Institute, Parkville, Melbourne, Victoria, Australia; 5 Department of Medical Oncology, Western Health, Footscray Hospital, Melbourne, Victoria, Australia; 6 Department of Surgery, Western Health, Level 3, WCHRE Building, Sunshine Hospital, 176 Furlong Road, St Albans, VIC 3021 Australia

**Keywords:** Body composition, Artificial intelligence, Rectal cancer, Sarcopenia, Survival outcomes

## Abstract

**Background:**

Sarcopenia has been widely studied in rectal cancer with increasing evidence to suggest that other body composition parameters, in particular adipose tissue, have an important role. Advances in artificial intelligence (AI) now allow 3D body composition analysis of intermuscular/intramuscular adipose tissue (IMAT) from CT scans. This study aimed to develop and evaluate a skeletal muscle score (SMS), utilising skeletal muscle (SM) and IMAT measurements, to predict treatment response and survival outcomes for rectal cancer patients.

**Methods:**

A retrospective analysis was performed on 226 patients with localised rectal adenocarcinoma treated at Western Health between 2013 and 2024. Body composition metrics, including SM and IMAT volume and density from the L1-S5 vertebral region, were extracted using validated AI software. A SMS (0–4) was developed to predict overall complete response (oCR). The primary endpoint was oCR, defined as pathological complete response or sustained clinical complete response for at least 3 years. Secondary outcomes included overall, cancer-specific, and disease-free survival.

**Results:**

An oCR was achieved in 25.7% of patients and was significantly associated with a lower MRI T stage, increased age at diagnosis, and a better SMS, whilst active smoking decreased oCR in a multivariable analysis. Patients with an SMS of zero had a 0% oCR rate, whilst patients with a SMS of four had oCR rate of 60%. A higher SMS correlated with improved overall, cancer-specific, and disease-free survival.

**Conclusion:**

The SMS is a novel, AI-derived body composition assessment that is strongly correlated with treatment response and survival in rectal cancer patients. This scoring system could provide clinicians with individualised risk stratification to enhance patient counselling.

**Supplementary Information:**

The online version contains supplementary material available at 10.1007/s00384-026-05106-w.

## Introduction

Skeletal muscle (SM) loss and sarcopenia have been central to body composition research in colorectal cancer over the past decade, with emerging evidence suggesting adipose tissue compartments also impact oncological and surgical outcomes [1, 2]. Advances in artificial intelligence (AI) and machine learning have transformed computed tomography (CT)-based body composition analysis from time-consuming 2D methods to automated 3D volumetric analysis [3].

Neoadjuvant treatment for rectal cancer has experienced a shift in practice with an increased uptake of total neoadjuvant therapy (TNT) for locally advanced rectal cancer (LARC) [4]. Landmark trial results have shown increased rates of pathological complete response (pCR) with this treatment paradigm [5, 6].

This study aimed to develop a novel method of skeletal muscle and intermuscular/intramuscular adipose tissue (IMAT) assessment utilising staging CT scans to predict treatment response, providing individualised risk stratification for LARC patients treated with neoadjuvant therapy.

## Materials and methods

A retrospective analysis (2013–2024) of rectal cancer patients was performed to evaluate the influence of skeletal muscle and adipose tissue volume and quality on treatment outcomes. Approval was granted by the Western Health Ethics Committee (project number HREC/24/WH/103022). This study adhered to the STROBE reporting guideline.

### Patient selection

Patients treated with rectal cancer at Western Health (WH) were identified from a prospective clinic registry, the Australian Comprehensive Cancer Outcomes and Research Database (ACCORD). Inclusion required a histological diagnosis of adenocarcinoma and to be treated with neoadjuvant therapy. Exclusion criteria included metastatic disease at diagnosis or a patient’s staging CT scan not being accessible.

### Data collection

The ACCORD registry was used to collect patient demographics, tumour biology, and oncological and surgical outcome data, which was cross-referenced with the WH electronic medical records. Body composition data was obtained from each patient’s staging CT scan prior to any treatment. Body composition analysis was performed with validated AI software capable of fully automated segmentation [3].

### Neoadjuvant oncological treatment

Patients with LARC at WH are treated with neoadjuvant therapy, which involves chemoradiotherapy (CRT). Long-course radiotherapy (50 Gray in 25 fractions) is given with a fluoropyrimidine agent (infusional 5-FU or capecitabine). TNT has been considered since 2020, with patients receiving neoadjuvant chemotherapy in addition to CRT. Four cycles of CAPOX or six cycles of FOLFOX are used during TNT treatment. The use of TNT versus long-course chemoradiation alone was decided at a multidisciplinary team (MDT) meeting.

### Endpoints

The primary endpoint was overall complete response (oCR), which has been defined as either pCR following surgical resection or clinical complete response (cCR) when sustained for greater than 3 years of surveillance as determined by the colorectal cancer MDT at WH. cCR was defined by the absence of a palpable rectal tumour, no visible tumour and a white scar on endoscopic assessment, and an MRI tumour regression grade (mrTRG) of 1–2 with no suspicious lymph nodes or extramural venous invasion following neoadjuvant treatment. A 3-year period of sustained cCR was used in the oCR definition as 99% of local regrowth, from residual tumour, is identified within 3 years of surveillance [7]. Secondary endpoints included overall, cancer-specific, and disease-free survival, neoadjuvant chemotherapy outcomes, and surgical outcomes.

### Body composition measurement

**Fig. 1:**
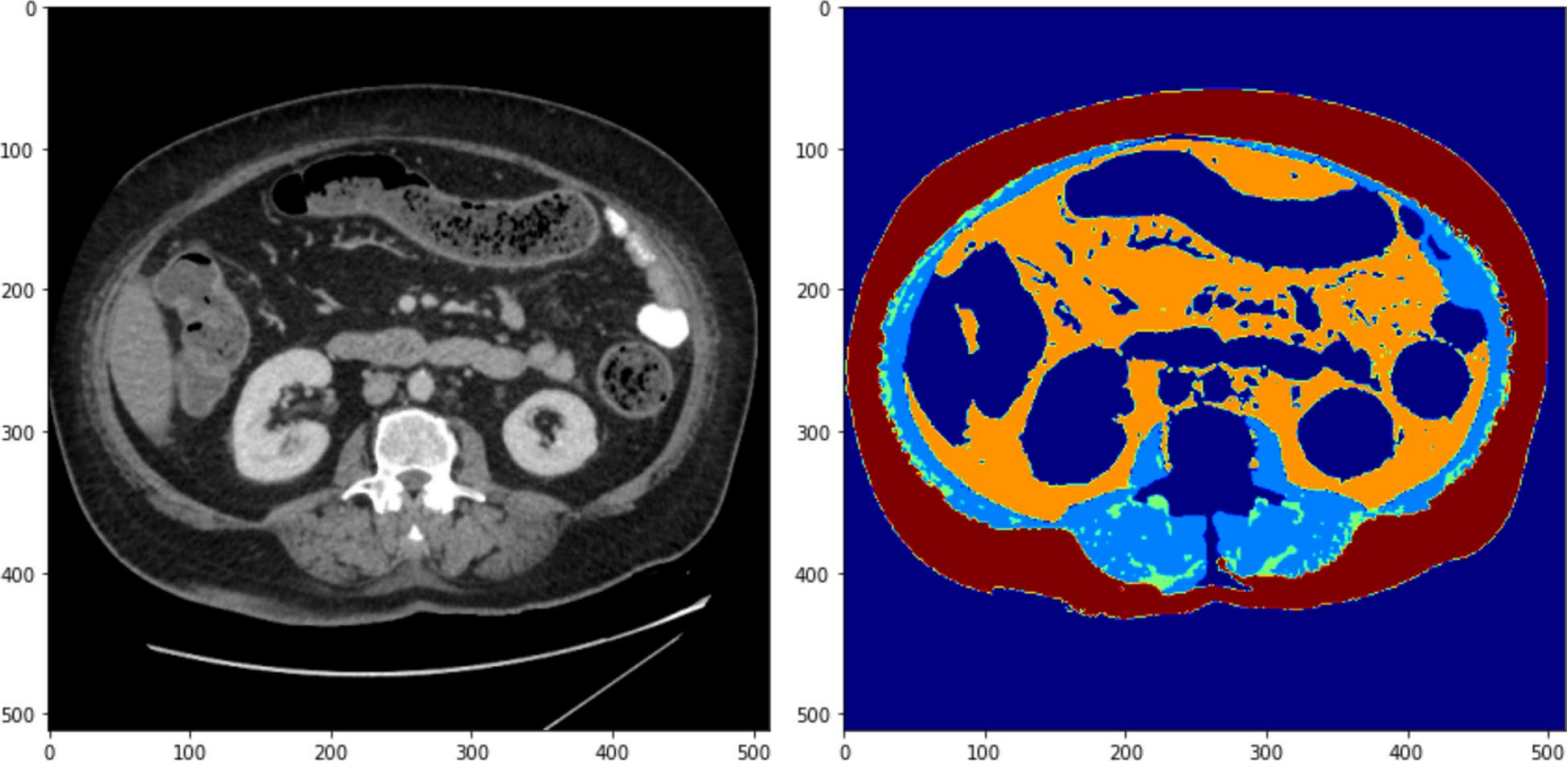
AI-generated body composition assessment of a single axial CT image. Light blue: skeletal muscle; green: intermuscular/intramuscular adipose tissue; orange: visceral adipose tissue; red: subcutaneous adipose tissue

Three-dimensional lumbosacral body composition was obtained from the analysis of diagnostic CT scans prior to neoadjuvant treatment. Tissue volume (cm^3^) and radiodensity (Hounsfield units, HU) values for SM and IMAT were calculated by a validated AI segmentation model [3]. All inhouse CT scans were performed using GE Healthcare. CT scans performed external to WH were transferred to the local hospital network at the time of diagnosis. External scans included additional manufacturers (Philips, Canon Medical Systems, and Siemens Healthineers). Inhouse CT scans were all performed with a slice thickness of 3 mm with a portal venous contrast series. External CT scans had a CT slice thickness ranging from 1 to 5 mm. Tissue area was multiplied by axial CT slice thickness for each axial image in the lumbosacral region to determine tissue volume. Average radiodensity was derived from the sum of the mean radiodensity in each axial slice divided by the number of slices in the lumbosacral region. A poor-quality CT scan or extension of soft tissue outside the captured CT image resulted in patients being excluded from analysis. A body composition assessment example from a single axial slice is shown in Fig. 1 and is repeated for all axial slices within the lumbosacral region.

SM volume index and IMAT volume index (cm^3^/m^2^) were calculated by dividing the volumetric values by patient height (metres) squared to allow for direct comparison. A skeletal muscle score (SMS) was developed from the SM and IMAT volume index and density values based on the optimal cutpoint for predicting oCR. A 5-tier system (0–4) in which a point was scored when a patient’s SM volume index, SM density, or IMAT volume index is above the identified cutpoint, or their IMAT density is below the cutpoint. This scoring system is data driven and based on each body composition metric’s ability to predict oCR. A score of zero denotes the ‘worst’ SMS.

### Statistical analysis

Baseline demographic characteristics were summarised and compared across study groups using appropriate descriptive measure. Distributional assumptions were evaluated through visual inspection of histograms. Continuous variables with a skewed distribution were reported as a median value with an interquartile range (IQR) and compared using the Mann–Whitney *U* test, whilst categorical data was analysed using Fisher’s exact test. The cohort was randomly allocated into a training set (*n* = 166, 73.5%) and an independent validation set (*n* = 60, 26.5%). Receiver operating characteristic (ROC) curves were generated for each body composition variable to estimate the corresponding area under the curve (AUC). These AUCs were used to quantify the association between each metric and oCR, and optimal threshold values were determined from the training cohort using the Liu method. Differences between the training and validation cohorts were assessed using Fisher’s exact and Breslow–Day tests. Multivariable logistic regression modelling was conducted to examine the association between oCR and SMS whilst adjusting for relevant clinical and treatment covariates. Survival outcomes were analysed using the Kaplan–Meier estimates with group comparisons performed via the log-rank test, and Cox proportional hazards models were applied with censoring for patients lost to follow-up. STATA (version BE 18.0) was used for all statistical analyses.

## Results

**Fig. 2:**
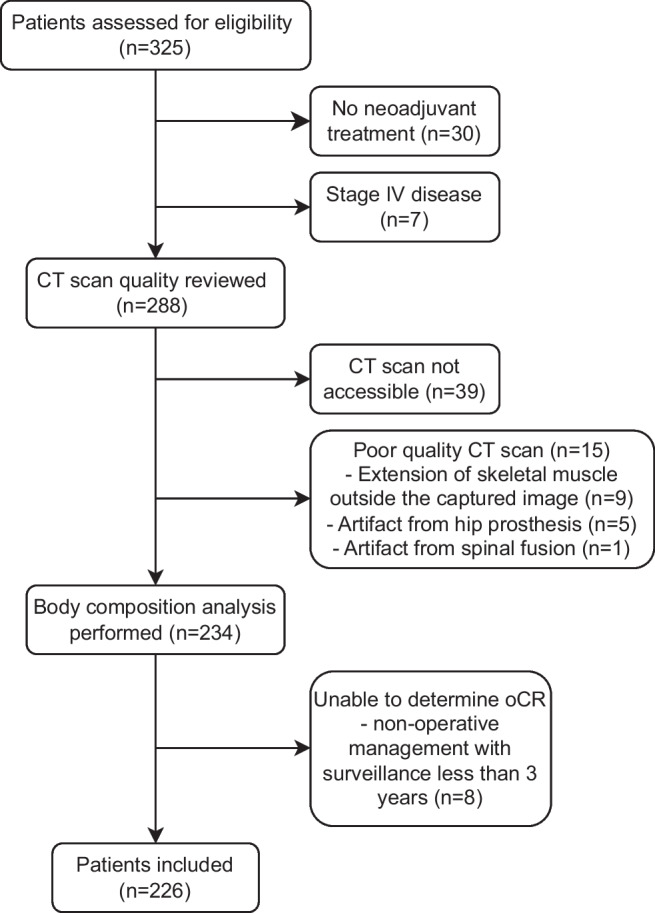
Patient inclusion/exclusion flow diagram

A total of 226 patients (male 156, 69%) diagnosed with rectal adenocarcinoma, treated with neoadjuvant therapy, at WH between 2013 and 2024 were included. Figure 2 outlines the reasons for excluding a further 99 patients. oCR occurred in 58 (25.7%) patients, which consisted of 48 postoperative patients with pCR and 10 patients managed non-operatively who have had sustained cCR for at least 3 years.

Table 1 summarises the cohorts’ baseline demographics and clinical characteristics and provides a subgroup analysis comparing patients with and without oCR. The oCR group was older (65.5 [11.8] vs 61.4 [12.9], *p* = 0.030) and had lower rates of current smokers with higher rates of lifetime non-smokers (10.3% vs 30.3% and 56.9% vs 38.7%, *p* = 0.004). There was no difference in the overall stage at diagnosis; however, a lower MRI T stage in patients that achieved oCR (T2: 29.8% vs 10.8%, T3: 61.4% vs 64.5%, T4: 8.8% vs 24.7%, *p* = 0.001) was observed.

**Table 1:** Baseline demographics and clinical characteristics

Parameter	Total (*n* = 226)	Overall complete response		*p*-value
		Yes (*n* = 58)	No (168)	
Age at diagnosis (years), mean (SD^a^)	62.5 (12.7)	65.5 (11.8)	61.4 (12.9)	**0.030**
Sex				0.869
Male	156 (69.0)	41 (70.7)	115 (68.5)	
Female	70 (31.0)	17 (29.3)	53 (31.6)	
Body mass index (kg/m^2^), mean (SD)	28.0 (5.6)	29.3 (5.6)	27.5 (5.3)	0.053
Body mass index category				0.413
< 18.5	4 (1.8)		4 (2.4)	
18.5–25	67 (29.7)	13 (22.4)	54 (32.1)	
25–30	84 (37.2)	24 (41.4)	60 (35.7)	
30–35	45 (19.9)	12 (20.7)	33 (19.6)	
> 35	26 (11.5)	9 (15.5)	17 (10.1)	
Smoking status				**0.004**
Never	98 (43.4)	33 (56.9)	65 (38.7)	
Ex-smoker	71 (31.4)	19 (32.8)	52 (31.0)	
Current smoker	57 (25.2)	6 (10.3)	51 (30.3)	
Diabetic status				0.402
- Non-diabetic	177 (78.3)	42 (72.4)	135 (80.4)	
Type I	1 (0.4)		1 (0.6)	
Type II	48 (21.2)	16 (27.6)	32 (19.0)	
ECOG^b^ score				0.819
0	179 (79.9)	45 (77.6)	134 (80.7)	
1	36 (16.1)	11 (19.0)	25 (15.1)	
≥ 2	9 (4.0)	2 (3.4)	7 (4.2)	
ASA^c^ score				0.520
1	11 (4.9)	4 (7.1)	7 (4.2)	
2	120 (53.8)	30 (53.6)	90 (53.9)	
3	90 (40.4)	21 (37.5)	69 (41.3)	
4	2 (0.9)	1 (1.8)	1 (0.6)	
Diagnosis T stage				**0.001**
T2	35 (15.7)	17 (29.8)	18 (10.8)	
T3	142 (63.7)	35 (61.4)	107 (64.5)	
T4	46 (20.6)	5 (8.8)	41 (24.7)	
Diagnosis N stage				0.214
N0	24 (10.8)	9 (15.8)	15 (9.1)	
N +	198 (89.2)	48 (84.2)	150 (90.9)	
Stage at diagnosis				0.143
I	12 (5.3)	6 (10.3)	6 (3.6)	
II	13 (5.8)	3 (5.2)	10 (5.9)	
III	201 (88.9)	49 (84.5)	152 (90.5)	
Distance to anal verge (mm), mean (range)	74.3 (0–158)	68.7 (0–138)	76.2 (0–158)	0.169
Long course chemoradiotherapy	226 (100)	58 (100)	168 (100)	0.331
Total neoadjuvant therapy	51 (22.6)	14 (24.1)	37 (22.0)	0.720
Treatment era^d^				0.540
< 2020	155 (68.8)	40 (69.0)	115 (68.5)	
≥ 2020	71 (31.4)	18 (31.0)	53 (31.5)	
Neoadjuvant treatment outcome				** < 0.001**
Residual disease	168 (74.4)		168 (100)	
pCR^e^	48 (21.2)	48 (82.8)		
Sustained cCR^f^	10 (4.4)	10 (11.2)		
Recurrence	53 (24.2%)	2 (3.5%)	51 (31.7%)	** < 0.001**
Local	9 (17.0)		9 (17.7)	
Distant	42 (79.2)	2 (100)	40 (78.4)	
Local and distant	2 (3.8)		2 (3.9)	
Overall death	37 (16.4)	4 (6.9)	33 (19.6)	**0.024**
Cancer specific death	20 (8.9)	1 (1.7)	19 (11.)	**0.030**
Duration of surveillance (years), median (IQR)	4.92 (2.32)	5.00 (2.13)	4.60 (2.38)	0.219

^a^Standard deviation

^b^Eastern Cooperative Oncology Group

^c^American Society of Anesthesiologists

^d^Treatment era based on the introduction of TNT to health network

^e^Pathological complete response

^f^Clinical complete response

Sex-specific body composition outcomes (Supplementary Table 1) showed similar values between oCR and non-responder groups. The IMAT volume index was greater (134 [65] vs 119 [86] cm^3^/m^2^, *p* = 0.036) in male patients with oCR when compared to those without oCR. Sex-specific cutpoint values for SM and IMAT volume index and density (Table 2) were determined from ROC analyses (Supplementary Table 2) based on the training dataset. Using these thresholds, the SMS was calculated for each patient (Table 3). The SMS distribution in the training and validation datasets showed no statistical difference (*p* = 0.267), and the SMS effect on oCR in each group was homogenous (*p* = 0.865). An increase in SMS was associated with an increase in oCR (*p* = 0.006). Patients with a SMS of zero had a 0% oCR rate compared to 60.0% for patients with a SMS of four. This trend was maintained for male patients (0% oCR for SMS of 0, 50% oCR for SMS of 4, *p* = 0.023) but did not reach significance for female patients (0% oCR for SMS of 0, 100% oCR for SMS of 4, *p* = 0.057). The SMS impact on oCR based on neoadjuvant treatment modality (CRT vs TNT) is detailed in Supplementary Table 3.

**Table 2:** Body composition cutpoint for the skeletal muscle score derived from training dataset

Body composition	Skeletal muscle scoring system	
	Male	Female
SM^a^ volume index (cm^3^/m^2^)	> 1650	> 1330
SM density (HU^b^)	> 39.5	> 38.6
IMAT^c^ volume index (cm^3^/m^2^)	> 125	> 129
IMAT density (HU)	< −57.9	< − 56.2

^a^Skeletal muscle

^b^Hounsfield units

^c^Intermuscular/intramuscular adipose tissue

**Table 3:** Muscle score and overall complete response rate

Muscle score	Training dataset (*n* = 166)		Validation dataset (*n* = 60)		Total cohort (*n* = 226)	
	Frequency	oCR^a^	Frequency	oCR	Frequency	oCR
0	10 (6.0%)	0%	4 (6.7%)	0%	14 (6.2%)	0%
1	56 (33.7%)	19.6%	12 (20.0%)	16.7%	68 (30.1%)	19.1%
2	58 (34.9%)	25.9%	29 (48.3%)	41.4%	87 (38.5%)	31.0%
3	35 (21.1%)	28.6%	12 (20.0%)	16.7%	47 (20.8%)	25.5%
4	7 (4.2%)	57.1%	3 (5.0%)	66.7%	10 (4.4%)	60%

^a^Overall complete response

**Fig. 3:**
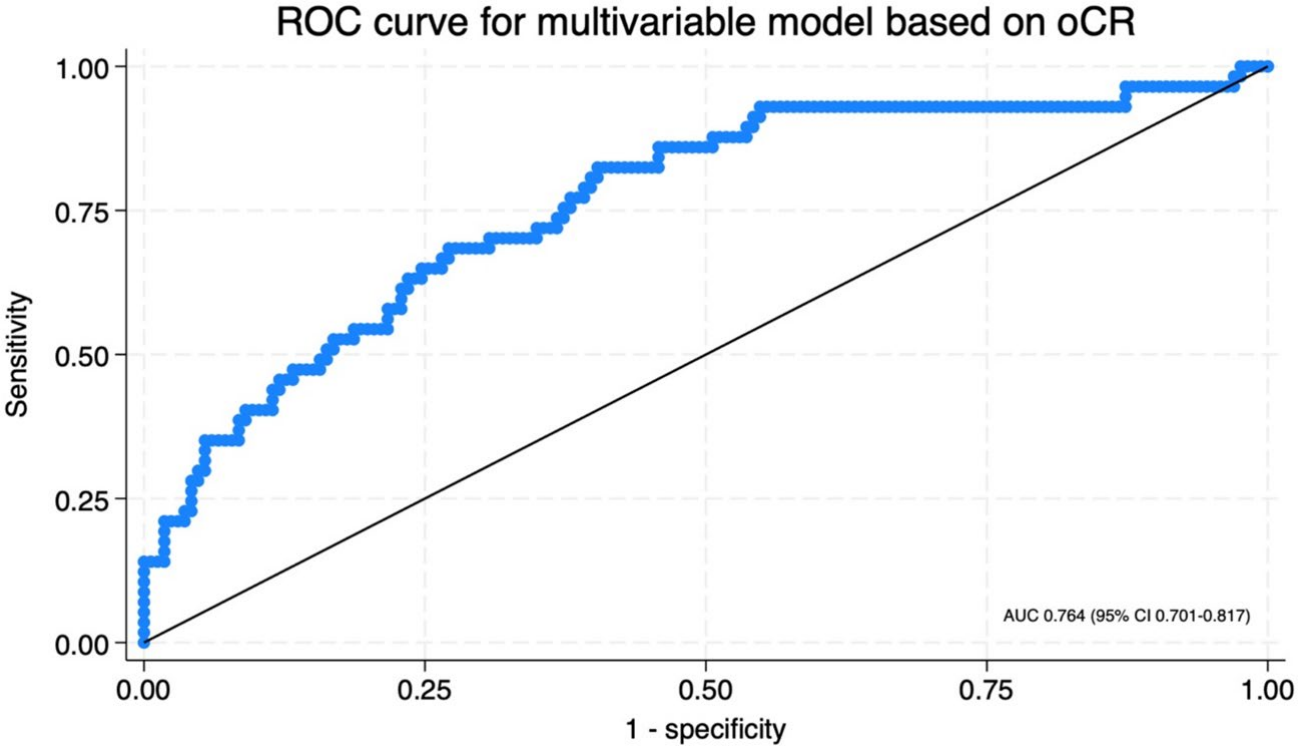
ROC curve for multivariable model based on overall complete response

Multivariable logistic regression (Table 4) identified MRI T stage to be associated with oCR with an odds ratio of 0.80 (95% CI 0.68–0.95, *p* = 0.010). Patients that were current smokers had a reduced incidence of oCR; odds ratio 0.50 (95% CI 0.29–0.84, *p* = 0.010). Increasing age had a small but significant impact on oCR, odds ratio 1.03 (95% CI 1.00–1.06, *p* = 0.049). The SMS was independently associated with oCR with an odds ratio of 1.56 (95% CI 1.08–2.26, *p* = 0.017). A ROC curve (AUC 0.764, 95% CI 0.701–0.817) for the multivariable model is shown in Fig. 3.

**Table 4:** Multivariable logistic regression for overall complete response (pseudo *r*^2^ = 0.155)

Variable	Odds ratio (95% CI)	*p*-value
Age at diagnosis	1.03 (1.00–1.06)	**0.049**
Sex	0.71 (0.33–1.54)	0.391
Smoking status	0.51 (0.32–0.83)	**0.007**
MRI T stage	0.80 (0.68–0.95)	**0.010**
MRI N stage	0.89 (0.76–1.04)	0.132
Total neoadjuvant therapy	2.25 (0.93–5.42)	0.070
Skeletal muscle score	1.56 (1.08–2.56)	**0.017**

Recurrence occurred in 53 (24.2%) patients during the surveillance period. The rate of recurrence in the oCR cohort (2, 3.5%) was significantly (*p* < 0.001) lower than those that did not achieve oCR (51, 31.7%). Both episodes of recurrence in the oCR cohort were due to distant metastatic disease in patients with pCR on initial resection. Recurrence location in the incomplete response group was predominately distant recurrence (40, 78.4%) with 9 (17.7%) episodes of local recurrence and 2 (3.9%) cases of both local and distant recurrence. Thirty-seven (16.4%) deaths were documented during followup, including patients with documented recurrence of rectal cancer. The incidence of survival events was significantly lower in the oCR cohort (Death: 6.9% vs 19.6%, *p* = 0.024; cancer-related death: 1.7% vs 11.3%, *p* = 0.030). Survival outcomes are detailed in Table 1.

**Fig. 4:**
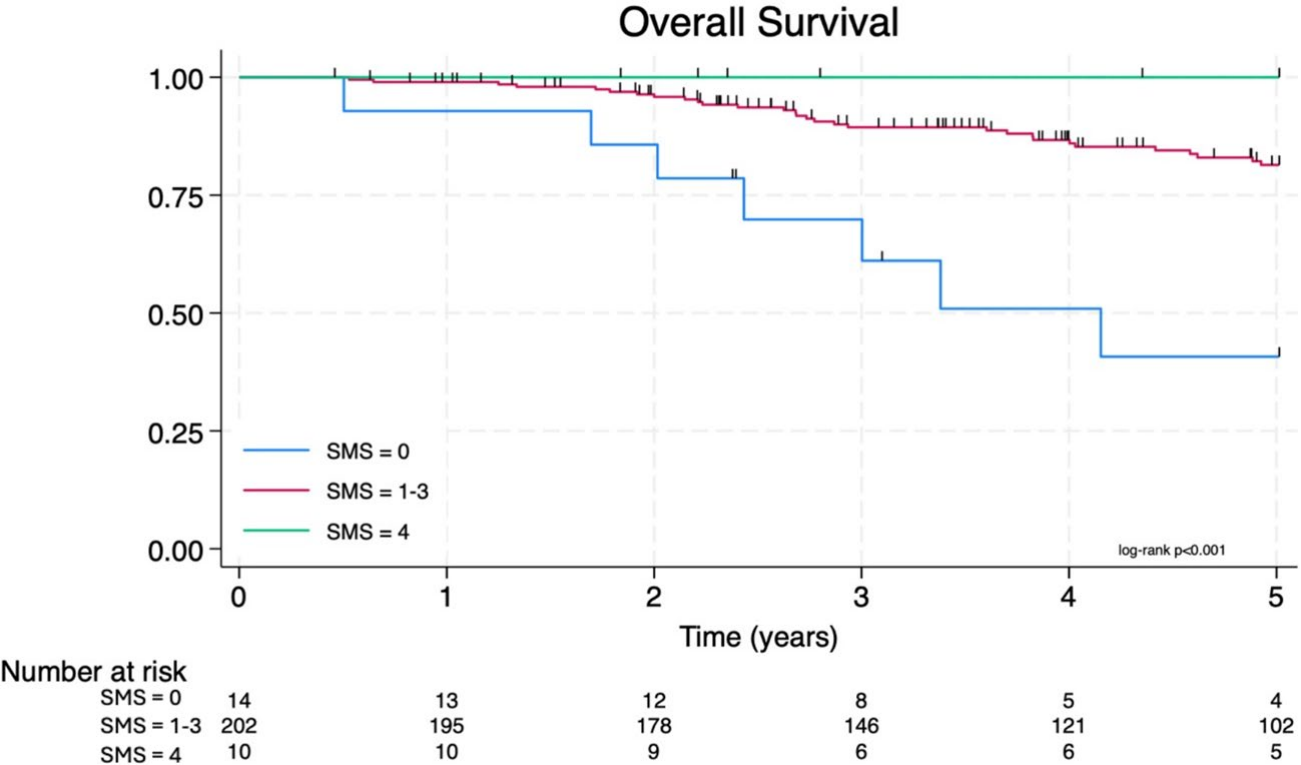
The Kaplan–Meier overall survival curve

**Fig. 5:**
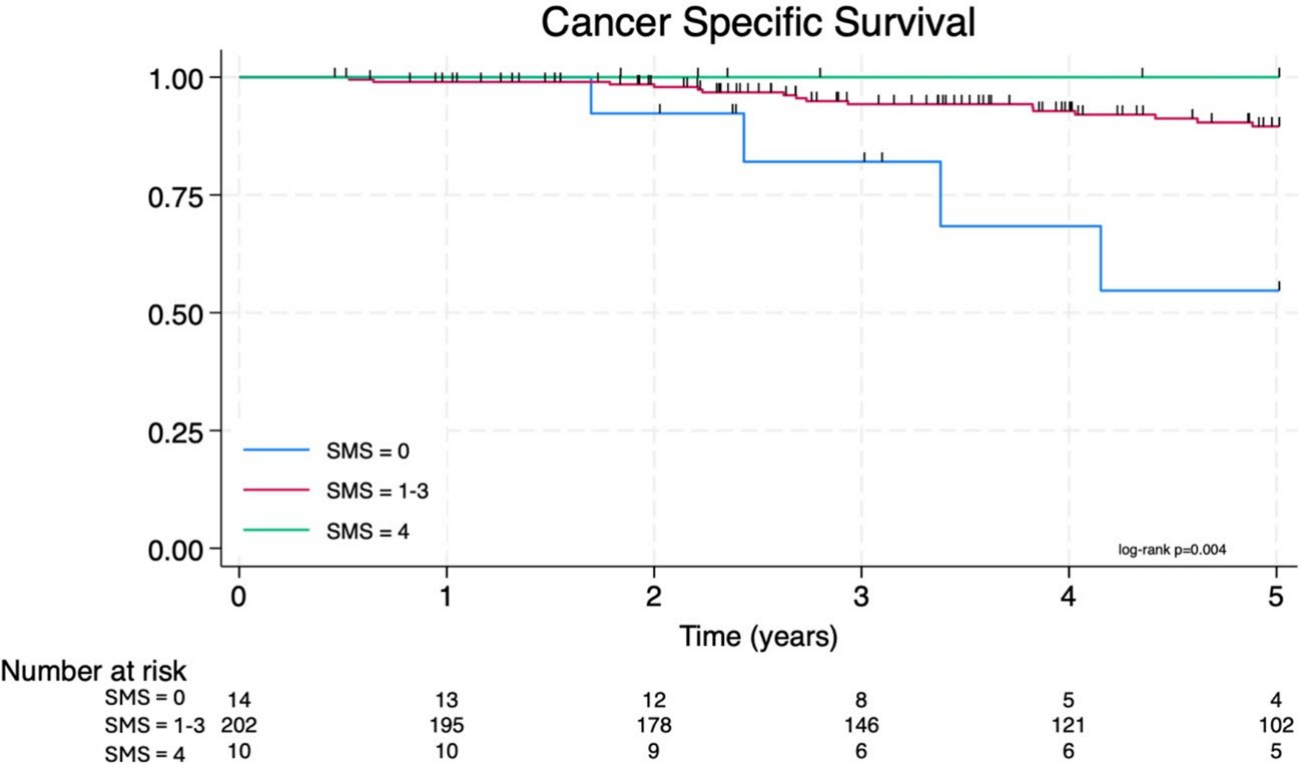
The Kaplan–Meier cancer-specific survival curve

**Fig. 6:**
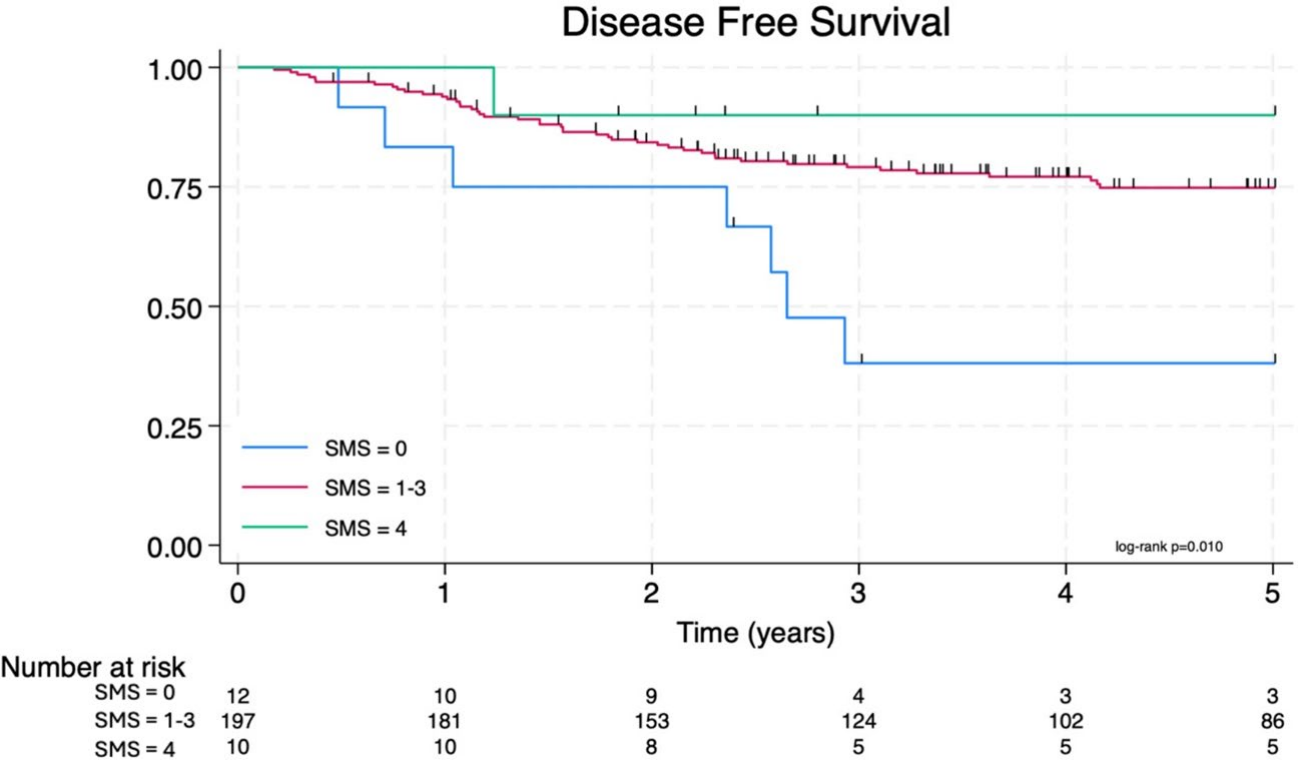
The Kaplan–Meier disease-free survival curve

Overall (*p* < 0.001), cancer-specific (*p* = 0.004), and disease-free survival (*p* = 0.010) decreased with a worse SMS. Kaplan–Meier curves for overall survival (Fig. 4) and cancer-specific survival (Fig. 5) both showed a 100% 5-year survival in the SMS four cohort. There was a significant reduction in all survival outcome measures with a reduced SMS, most notable in the SMS zero cohort. Patients with a SMS of 1–3 had similar survival outcomes and have been grouped in the analysis since this range of scores did not differentiate patients. A Cox proportional hazard model was performed for each survival metric with the SMS and key prognostic factors (Table 5). The SMS is an independent prognostic factor for overall survival (HR 0.28 [0.11–0.67], *p* = 0.004), cancer-specific survival (HR 0.21 [0.06–0.67], *p* = 0.009), and disease-free survival (HR 0.28 [0.12–0.66], *p* = 0.003) (Fig. 6). All other variables were not associated with survival outcomes except MRI T stage for disease-free survival (HR 2.24 [1.35–3.70], *p* = 0.002).

**Table 5:** Cox proportional hazard ratio for overall, cancer specific and disease-free survival

	Overall survival		Cancer specific survival		Disease-free survival	
	Hazard ratio (95% CI)	*p*-value	Hazard ratio (95% CI)	*p*-value	Hazard ratio (95% CI)	*p*-value
SMS^a^	0.28 (0.11–0.67)	**0.004**	0.21 (0.06–0.67)	**0.009**	0.28 (0.12–0.66)	**0.003**
Age at diagnosis	1.02 (0.99–1.05)	0.297	1.00 (0.97–1.04)	0.831	0.98 (0.96–1.01)	0.201
Sex	1.23 (0.59–2.56)	0.581	1.36 (0.53–3.48)	0.519	0.96 (0.52–1.79)	0.909
Smoking status	1.08 (0.68–1.71)	0.748	0.83 (0.45–1.54)	0.561	0.75 (0.52–1.07)	0.115
MRI T Stage	0.89 (0.49–1.60)	0.689	1.11 (0.51–2.41)	0.795	2.24 (1.35–3.70)	**0.002**
MRI N Stage	2.04 (0.47–8.87)	0.340	0.95 (0.21–4.41)	0.951	0.44 (0.19–1.03)	0.060
TNTb	2.38 (0.93–6.04)	0.069	0.91 (0.19–4.34)	0.906	1.13 (0.55–2.31)	0.742

^a^Skeletal muscle score

^b^Total neoadjuvant therapy

## Discussion

Our proposed skeletal muscle score is a novel grading system designed to combine skeletal muscle and IMAT quantity and quality to enhance risk stratification for rectal cancer patients undergoing curative treatment. We identified a strong correlation between SMS with oCR and survival outcomes. Patients with the best SMS of four had a 60% oCR rate, which dropped to 0% for those with the worst score (SMS 0). The SMS remained a significant predictor of oCR on multivariable regression analysis (*p* = 0.017), along with smoking status (*p* = 0.007), MRI T stage at diagnosis (*p* = 0.010), and age at diagnosis (*p* = 0.049).

Sarcopenia has consistently been shown to negatively impact response to neoadjuvant rectal cancer treatment. Lower pCR rates are seen in both CRT and TNT treatment pathways for sarcopenic patients [8, 9]. The underlying mechanism driving these differences remains unclear and is not due to lower chemotherapy exposure, as fluoropyrimidines and platinum-based chemotherapy agents are hydrophilic and primarily distributed in lean muscle. This means sarcopenic patients receive a higher chemotherapy dose because of a reduced volume of distribution [10], which could be one driver of increased adverse events.

The association between adipose tissue area and rectal cancer treatment response appears to be location dependent. There is limited evidence evaluating the impact of visceral adipose tissue (VAT) volume on pCR rates. However, increased mesorectal fat volume, which is a component of VAT and associated with total VAT volume, has been shown to increase pCR [11]. A greater mesorectal fat volume is associated with reduced nodal involvement, which could account for the improved rates of pCR [2]. The mechanism responsible for this is thought to be a combination of a buffering effect from the fat itself and from adipokines secreted from adipocytes, acting to inhibit local invasion and nodal metastasis [2]. Conversely, a high subcutaneous adipose tissue (SAT) area following neoadjuvant treatment for LARC has been associated with poorer tumour response and a lower histological tumour regression grade [1]. Chemotherapy resistance secondary to systemic inflammation mediated by cytokines released from SAT is one potential explanation for this finding [1].

Within the body composition literature, myosteatosis (intermuscular/intramuscular fat) has been traditionally reported as a low SM density. A low SM density has been used as a surrogate marker for IMAT volume/myosteatosis [12, 13] due to the lack of technology being able to accurately identify IMAT with precise detail. Although a correlation between low SM density and increased IMAT area has been established [14], the literature reporting 3D IMAT volume is sparse, and the direct influence of IMAT volume on pCR is unknown. Through machine learning, the advent of AI-derived body composition analysis now allows accurate discrimination of IMAT volume and density as a separate metric to SM [3]. Our results have shown that a higher IMAT volume index (134 vs 119cm^3^/m^2^, *p* = 0.036) was observed in male patients with oCR. Values below the identified sex-specific IMAT volume index cutpoint (male: 125 cm^3^/m^2^, female: 129cm^3^/m^2^) have been considered to place patients at an increased likelihood of poor treatment response when developing the SMS. The mechanism which underpins this finding is unclear, and the directional effect of IMAT volume results in predicting oCR was unexpected. Myosteatosis has not previously been associated with rectal cancer neoadjuvant therapy treatment response. A recent literature review identified 10 articles that examine the impact of myosteatosis on rectal cancer neoadjuvant therapy without any significant findings identified [15]. A possible explanation for this finding could be that IMAT volume represents a proxy for overall energy reserve, and an increased volume could counter the inherent cachexia driving factors associated with malignancy, which is known to impair tumour response to chemotherapy [16].

Myosteatosis is the accumulation of adipocytes in SM, which impairs muscular function. This accumulation of IMAT can occur within a muscle, between individual fibres (intramuscular), or between adjacent muscle groups bound by deep fascia (intermuscular). Muscle satellite cells, a stem cell responsible for muscular regeneration and growth, can develop an adipogenic phenotype in response to various stimuli and are responsible for the development of myosteatosis [17]. Pathological processes including obesity, hyperglycaemia, and hypoxia, as well as a sedentary lifestyle and ageing, can all result in adipogenic conversion of muscle satellite cells to increase intermuscular and intramuscular adipose tissues [17].

Myosteatosis has been shown to be associated with poor overall and cancer-specific survival as well as with increased postoperative complications such as anastomotic leak [18, 19]. Increased systemic inflammation, as demonstrated by an increased neutrophil-to-lymphocyte ratio (NLR), is seen in patients with myosteatosis [20]. There are many proposed mechanisms whereby an increased inflammatory state acts to promote tumour progression. These include an elevated level of pro-inflammatory cytokines, such as IL-6, which activate cellular signalling pathways to enable invasion and increase angiogenesis through elevated vascular endothelial growth factor [21].

Adipose tissue density increases due to fibrosis following periods of rapid weight loss or from inflammatory and metabolic changes from proinflammatory cytokines that are released from the tumour itself [22–24]. Increased visceral and SAT density is well established to be associated with decreased overall survival in colorectal cancer as well as other cancer types [25, 26]. There is a lack of published data looking at the specific correlation of IMAT density on survival outcomes or tumour response. However, it is assumed that IMAT density would also increase to the same systemic stimuli that drive visceral and SAT density changes in colorectal cancer. For these reasons, we have assessed IMAT density values greater than the sex-specific cutpoint threshold (male: −57.9 HU, female: −56.2 HU) to place patients at increased risk.

Smoking was shown to be associated with a twofold reduction in the rate of oCR on multivariable analysis. Chuang et al. have recently reported the same twofold risk increase for a poor tumour regression grade amongst active smokers with LARC treated with CRT [27]. Multiple cellular pathways can explain the association between smoking and reduced tumour response. For example, smoking results in systemic and tumour hypoxia; this reduces the impact of ionising radiation [28] and the effectiveness of chemotherapy agents due to a reduction of apoptosis signalling pathways [29]. Increased age at diagnosis was identified as a weak but significant positive predictor of oCR on multivariable regression. This finding is in keeping with previous publications, including a meta-analysis with 6725 patients [30, 31].

In patients with locally advanced gastric cancer treated with neoadjuvant chemotherapy and gastrectomy, a two-parameter SMS (SM density and change in skeletal muscle index) proposed by Lin et al. was strongly predictive of pathological response [32]. Similarly, Cheng et al. have developed a body composition scoring system for breast cancer patients comprising SM index, SAT index, and SAT density, which is predictive of mortality [33]. To our knowledge, there are no previous body composition scoring systems developed in colorectal cancer patients. However, sarcopenia and low SM density have been shown to have a joint effect that is synergistic and predicts worse outcomes in colorectal cancer patients when both are present versus either metric in isolation [13].

The survival outcomes reported in our study match those from recently published datasets [34, 35]. The SMS was significantly associated with overall, cancer-specific, and disease-free survival, with a better SMS being associated with better survival outcomes. Given that sarcopenia and myosteatosis have consistently been shown to decrease overall and cancer-specific survival, a worse SMS was expected to be associated with poor long-term outcomes [36, 37].

Rectal cancer involves a long period of neoadjuvant therapy, particularly during TNT, allowing for patient intervention and optimisation. Not only do negative body composition traits favour poor outcomes but also further deterioration in SM mass during neoadjuvant treatment is associated with reduced disease-free survival [38]. A prehabilitation study in rectal cancer patients has shown that an increase in muscle mass is possible during neoadjuvant therapy for those managed with telephone-guided exercise programs when compared to controls [39]. Given this, large-scale trials to determine the feasibility and benefit of prehabilitation interventions are worth pursuing.

There is also potential for the SMS score at diagnosis to inform multidisciplinary clinic decision-making. For patients with a SMS of zero or four, there is a 100% sensitivity and 78% specificity in predicting oCR within the total cohort. For patients treated with TNT, the sensitivity remained 100%, whilst specificity slightly decreased to 75%. Given this, the SMS score could inform a discussion regarding the use of chemoradiation versus TNT, including for older/frailer patients, and help to set patient expectations. For patients achieving a cCR, deciding between initial non-operative management versus surgical resection could also be informed by the SMS.

For future clinical trials of neoadjuvant therapy strategies, the SMS score at diagnosis could be a useful stratification factor, given that the association with outcomes is as strong or stronger than for conventional stratification factors, such as T and N stage.

### Limitations

Despite internal validation and the strong correlation SMS has with oCR and survival in our study, our novel scoring system will need to undergo external validation in an independent series to confirm the robustness and reproducibility of our findings prior to use in a clinical setting. There are multiple oncological neoadjuvant treatment pathways (CRT and TNT) in our patient cohort; although this variable has been considered in our multivariable regression, this could bias survival outcomes. Since TNT has become the standard of care for patients with LARC, external validation testing of the SMS should be performed in a cohort of patients treated with a TNT protocol. Although WH has a multicultural patient population, further ethnicity-based assessment should be conducted given the known body composition variation between different ethnic groups. We have also assumed in our discussion that IMAT density will increase in response to the same systemic inflammatory stimuli present in LARC patients that drive visceral and subcutaneous adipose tissue density changes. A low IMAT volume index has been considered unfavourable in the SMS given the association found in male patients with oCR; however, the underlying mechanism to explain this finding is unclear and is a focus of future research.

## Conclusion

Our novel SMS can predict oCR and survival outcomes in LARC patients treated with neoadjuvant therapy. Use of this body composition scoring system could be used in future clinical settings to help with risk stratification.

## Supplementary Information

Below is the link to the electronic supplementary material.

ESM1 (DOCX 19.4 KB)

ESM2 (DOCX 215 KB)

## Data Availability

Data is provided within the manuscript and supplementary information files.
