# Supplementary material for: Utilisation of intramuscular and intermuscular fat to develop a new skeletal muscle grading score which can predict treatment outcomes for locally advanced rectal cancer
Source: Int J Colorectal Dis. 2026 Feb 16;41(1):65. doi: 10.1007/s00384-026-05106-w (PMC12907271; doi:10.1007/s00384-026-05106-w)
Supplement: Supplementary file 1 — (DOCX 19.4 KB) [file 384_2026_5106_MOESM1_ESM.docx]

**Supplementary Material**

Supplementary Table 1: Sex specific univariate analysis of body composition metrics with overall complete response

| Body composition | Male | | p-value | Female | | p-value |
| --- | --- | --- | --- | --- | --- | --- |
|  | oCR^a^ | Incomplete response |  | oCR | Incomplete response |  |
| SM^b^ Index (cm^3^/m^2^), median (IQR^c^) | 1714 (296) | 1725 (466) | 0.656 | 1394 (431) | 1294 (343) | 0.301 |
| SM Density (HU^d^), median (IQR) | 40.4 (10.4) | 41.0 (12.2) | 0.204 | 38.7 (14.4) | 38.5 (12.0) | 0.765 |
| IMAT^e^ Index (cm^3^/m^2^), median (IQR) | 125 (91) | 100 (78) | **0.036** | 132 (135) | 97 (79) | 0.073 |
| IMAT Density (HU), median (IQR) | -57.0 (3.0) | -56.4 (4.8) | 0.242 | -56.1 (8.4) | -54.9 (5.6) | 0.190 |

a: Overall complete response, b: Skeletal Muscle, c: Interquartile range, d: Hounsfield Units, e: Intramuscular/intermuscular Adipose Tissue

Supplementary Table 2: Gender specific AUC values from ROC curve based on body composition prediction of overall complete response

| Body composition | Area under the ROC^a^ curve (95% CI) | |
| --- | --- | --- |
|  | Male | Female |
| SM^b^ volume index (cm^3^/m^2^) | 0.485 (0.370-0.599) | 0.557 (0.379-0.735) |
| SM density (HU^c^) | 0.442 (0.318-0.565) | 0.452 (0.270-0.635) |
| IMAT^d^ volume index (cm^3^/m^2^) | 0.593 (0.471-0.715) | 0.641 (0.460-0.823) |
| IMAT density (HU) | 0.478 (0.359-0.596) | 0.430 (0.255-0.606) |

a: receiver operating characteristic, b: Skeletal Muscle, c: Hounsfield Units, d: Intermuscular/intramuscular Adipose Tissue

Supplementary Table 3: Skeletal muscle score and overall complete response rate based on neoadjuvant treatment modality.

| Muscle score | CRT (n=175) | | p-value | TNT (n=51) | | p-value |
| --- | --- | --- | --- | --- | --- | --- |
|  | Frequency | oCR^a^ |  | Frequency | oCR |  |
| 0 | 11 (6.3%) | 0% | 0.112 | 3 (5.8%) | 0% | 0.092 |
| 1 | 56 (32.0%) | 21.4% |  | 12 (23.5%) | 8.3% |  |
| 2 | 67 (38.3%) | 29.9% |  | 20 (39.2%) | 35.0% |  |
| 3 | 35 (20.0%) | 25.7% |  | 12 (23.5%) | 25.0% |  |
| 4 | 8 (3.4%) | 50.0% |  | 4 (7.8%) | 75.0% |  |

a: Chemoradiotherapy, b: Total neoadjuvant therapy, c: Overall complete response
